# Investigation of importance of the structural parameters of the eyeball and of the technical parameters of cataract surgery on corneal endothelial changes

**Published:** 2018

**Authors:** Razvan Vladimir Nanu, Emil Ungureanu, Sinziana Luminita Istrate, Alexandra Vrapciu, Roxana Cozubas, Laura Carstocea, Liliana Mary Voinea, Radu Ciuluvica

**Affiliations:** * “Sf Ioan” Emergency Hospital, Bucharest, Romania; ** Ophthalmology Department, University Emergency Hospital; “Carol Davila” University of Medicine and Pharmacy, Bucharest, Romania; *** ”Grigore Alexandrescu” Emergency Hospital for Children, Bucharest, Romania; **** PhD Student, “Carol Davila” University of Medicine and Pharmacy, Bucharest, Romania; ***** Anatomy Department, “Carol Davila” University of Medicine and Pharmacy, Bucharest, Romania

**Keywords:** cataract surgery, phacoemulsification, endothelial cell density, viscoelastic

## Abstract

**The aim of the here presented study** was to look into the importance of the structural parameters of the eyeball, in relationship with the technical parameters of cataract surgery regarding the corneal endothelial changes made by it.

**Material and method:** The paper refers at a prospective study in which we included eighty-eight consecutive eyes from eighty-six different patients having age-related cataract and a visual acuity of a values less than 0.8 considering best possible correction with it. The patients didn’t have other obvious causes for the decreased visual acuity. The patients included in the present study were admitted at the Ophthalmology Department from Bucharest University Emergency Hospital between the month of April 2015 and February 2016 (ten months).

**Results:** When we compared lots A, B with C, in regarding to the decrease of EDC, the results were relatively very similar. We had only one comparison for which we obtained a statistical significance, and that was for cataracts classified as group IV of hardness; here, between the first and the third lot, at seven days postoperatively we obtain p = 0.0472812.

**Conclusions:** The conclusion for the present research was that in regarding cataract phaco-emulsification surgery we obtained a statistical significance when it comes to the destruction of epithelial cells. The results were observed, giving the depth of the anterior chamber, in cataracts classified in subgroup IV of hardness, only between patients who had a small depth of chamber comparing with those who had a large depth of the anterior chamber of the eye. When it comes to patients who had severe cataracts and small ACD, we need to attract more attention when the surgeon performs the maneuver and to keep an eye on the use of the adhesives which has viscoelastic in order to obtain additional corneal protection.

**Abbreviations**:

ACD = anterior chamber depth; ECD = endothelial cell density, EPT = effective time of phacoemulsification.

**The purpose of the present study**

The purpose of the here presented study was to search the importance of anatomical, structural and constitutional parameters of the eyeball, when it comes to technical parameters in relationship with cataract surgery at the level of corneal endothelial variations and changes. This goal was brought into light by the fact that now there is no consensus in the present literature, on the need for differential approach of the cases, if we take into consideration and according to these parameters; in present, literature is insufficiently standardized and systematized [**1**-**5**]. 

Even today, cataract surgery remains the most accepted common surgical procedure [**6**] even in countries with advanced economies. Of course, in the last years, the procedure benefited from significant progresses, in regarding the technology but also in surgical technique, leading to superior outcomes, a rapid and increased postoperative recovery after the surgery, and a significant decrease concerning the complication rates. 

## Material and method 

The here presented study was a prospective type that included 88 consecutive eyes from 86 different patients who had age-related cataracts and having less than 0.8 visual acuity with the best possible correction, without other obvious causes of decreased visual acuity. The patients were admitted to the Ophthalmology Department of Bucharest University Emergency Hospital between April 2015 and February 2016.

**Inclusion criteria for the patients admitted in this study:**

- patients who had over 18 years of age, to whom we have explained the need in regarding the intervention, if there were possible variants at the time and patients who were able to sign an informed consent at the time of enrolling;

- patients who experienced age-related cataract, grades 2-4, with ACD above 1.5 mm;

- patients with medically controlled open-angle primitive glaucoma;

- intraocular pressure below 21 mm Hg without treatment;

- in regarding of the distribution of patients with gender, we didn’t observe the proportion, we enrolled consecutive patients in the here presented study;

- our patients were race type Caucasians, but this wasn’t a criteria that we followed for inclusion, but a regular distribution of the population in Romania.

**Exclusion criteria**

- patients who had other types of cataract than the one observed and studied here - age-related cataract;

- we had patients who had Fuchs corneal dystrophy, we took them out from here, but they were admitted in a sub-study to be presented later;

- patients who had some sort of history of ocular inflammatory disease;

- patients with ECD running below 1800 cells/ mm2;

- when it comes to intraocular pressure, the patients who had it greater than 21 mmHg at the time of presentation were excluded from the present study;

- patients with anterior-eye surgery;

- patients who turned out to be with complicated cataract surgery – those whom we thought that it was necessary to implant capsular tension rings into the bag of the lens;

- patients who didn’t follow the rules regarding the ulterior visits were also eliminated from the study.

**Objectives of the present study**

We had a main objective in regarding the study which was to spot a possible relationships between the structural and anatomical parameters of the eyeball (ACD, crystalline hardness, crystalline lens, ECD prior to the surgery, pachymetry), technical parameters regarding the cataract surgery (ETP) and post-operative structural modification at various intervals of ECD respectively.

Secondary endpoints were to determine a relationship which can appear between the above-mentioned structural and anatomical parameters and the recovery of visual acuity in the postoperative period.

**Initial examination**

The initial examination was conducted using anamnesis, a wide general exam, a visual acuity measurement, examination of the anterior pole and the fundus, biometry, pachymetry, intraocular pressure determined by aplanotonometry, keratometry, a specular microscopy.

**Visual acuity** was taken with a Snellen optotype.

**Aplanotonometry** was performed in order to obtain intraocular pressure, with the Goldman aplanotonometer. Measurements were made using topical anesthesia with 4% oxy-buprocaine hydro-chloride. All intraocular pressure measurements were made by the same medical staff in order to ensure good reproducibility when it comes to different patients as well as when it comes to different measurements made for the same patient. The results were rounded, and we noted the nearest full intraocular pressure (we didn’t use fractional values).

**Biometry** was achieved with a biometer Ocu Scan (Alcon), by immersion, using topical anesthesia made with 4% oxy-buprocaine hydro-chloride. We took ten valid measurements for each of the patient.

**Keratometry** was made with the Topcon autorefractometer.

**Pachymetry** was performed with the Omac Scan (Alcon) – a biometric probe - it was also checked using a specular microscope Topcon. We took ten valid measurements for each patient and we monitored the central thickness of the cornea.

**Specular microscopy** was investigated with a specular microscope from Topcon. We took three central measurements for each of the patient. We marked the center of cells for 50 cells in the endothelial cornea in each case. In this measurement, we monitored the ECD, the percentage of the cells and their distribution as a size. The endothelial cell loss was counted using the formula: (initial endothelial cells - postoperative endothelial cells)/ (initial endothelial cells x 100).

**Surgical technique**

Phacoemulsification was achieved in all cases in the hand of the same surgeon. Mydriasis was made by alternative administration of Tropicamide in concentration of 1% and also Phenylephrine 10%. Anesthesia was a topical one, made by administrating of 4% oxy-buprocaine hydro-chloride and a gel 4% tetracaine. We performed two corneal contra-incisions in the clear corneas at hours ten and two using a pre-calibrated diamond blade of 1.2. For each lot, half of the interventions were done by coaxial technique and half by bimanual technique.

For the **coaxial technique**, the main incision was in the clear cornea, triplanar, with diamond blade pre-incision, then biplanar with a 2.2 mm knife. Cohesive viscoelastic was injected into eyeball (the anterior chamber). A continuous circular capsulorhexis with a cystotome and a Duckwort & Kent capsulorhexis clip type Inamura was performed. The hydro-dissection and the hydro-delineation were performed using a 26 G cannula. The phacoemulsification was made using the technique named phaco chop. The technique of the irrigation/ aspiration was executed bimanually. After insertion of the pseudophakia into the bag, viscoelastic aspiration and wound hydro suture were performed.

In the **bimanual technique**, phacoemulsification was achieved using a phaco-tip without a sleeve and the irrigation chopper placed on the two 1.2 mm paracentesis. Capsulorhexis was made with a Kershner One-Pinch capsulorhexis clip. Otherwise, the technique of the surgery was similar. At the end of the surgery, we inserted viscoelastic into the bag and also into the eye (anterior chamber). We chose one of the contra-incisions in order to minimize the astigmatism and we widened it to 1.8 mm in order to introduce the foldable lens in the bag.

**Postoperative treatment** was conducted with drops of indometacin and a fixed combination of betamethasone and chloramphenicol qid for 7 days, then three times per day for another 3 weeks. We called back the patients at one day, one week, 4 weeks, 3 months, in order to check parameters: pachymetry, ECD, visual acuity. 

**Distribution on lots**

In total, we observed 88 eyes with age-related cataract (the nuclear density was between 2 and 4); patients met the inclusion conditions in the study. Subsequently, two of the patients didn’t show up at the moment indicated for the postoperative controls; therefore, they were excluded from the present study.

We divided the patients into 3 lots depending on the ACD. Lot A presented patients with preoperative ACD between 1.5 mm and 2.3 mm. Lot B showed patients with the ACD between 2.3 mm and 3.5 mm. Lot C showed patients with a preoperative ACD of more than 3.5 mm.

**Lot A** was composed of patients with ACD between 1.5 mm and 2.3 mm. 19 patients were included here in this group (21.59% of the patients). Of these, 7 patients experienced grade II cataract (36.84%), 9 patients grade III (47.36%) and 3 of them had grade IV (15.78%).

**Lot B** was accomplished from patients with ACD between 2.3 mm and 3.5 mm. 51 patients (57.95%) were part of this group. Of these, 16 patients had grade II cataract (31.37%), 24 patients grade III (47.05%), and 11 patients grade IV (21.56%).

**Lot C** was made up of patients with an ACD of more than 3.5 mm. 18 patients (20.45%) were included in this group. Of these, 7 patients experienced grade II cataract (44.44%), 9 patients grade III (47.36%) and two patients grade IV (10.52%).

## Results

The table and figures below show the distribution of patients by age group and gender distribution.

**Fig. 1:**
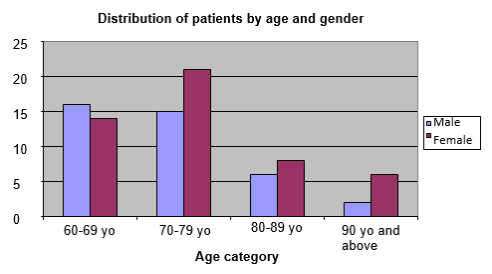
Distribution of patients by age and gender

There were 39 male patients (44.31%) and 49 female patients (55.68%). When it comes to age distribution, we observed that the patients were predominantly in the age group from 60 to 69 years of age (30 patients, 34.09%) and in the age group 70-79 years (36 patients, 40.90) and less in the 80-89 age groups (14 patients, 15.9%) and 90 years or over (8 patients, 9.09%) (**Fig. 1**).

Following controls, for **lot A**, the following data was recorded - **Table 1**.

**Table 1:** Patients in group A. Patients with * were operated by bimanual technique

Patient	Sex	Cataract grade	ECD initial	ECD at 7 days	ECD at 1 month	ECD at 3 months	EPT
B.I. *	F	II	2207	2184	2189	2192	2.6
A.M.	M	III	2307	2154	2148	2199	4.1
G.V.	M	III	2512	2488	2507	2520	3.3
Z.A.*	F	IV	1909	1756	1824	1819	7.3
R.O.*	F	III	2088	1980	2020	2036	5.4
A.P.	F	II	2166	2145	2157	2155	1.6
M.B.*	M	III	1987	1912	1923	1945	2.8
L.P.	M	II	2735	2722	2710	2715	1.8
M.A.*	F	III	2267	2146	2156	2175	4.2
U.R.	M	III	2187	2073	2162	2178	3.4
V.M.*	F	IV	2275	1966	2124	2155	5.9
M.N.	F	II	2307	2085	2172	2248	3.7
P.P.	F	III	2569	2443	2453	2449	3.7
R.O.	M	II	2005	1988	1945	2026	1.4
S.T.*	F	III	2400	2210	2315	2324	4.2
T.F.	F	II	2333	2245	2259	2281	2.3
R.B.	M	IV	2154	1987	2009	2076	3.3
C.B.*	F	II	2763	2667	2641	2689	1.5
V.C.*	M	III	2343	2189	2233	2251	2.7

**Fig. 2:**
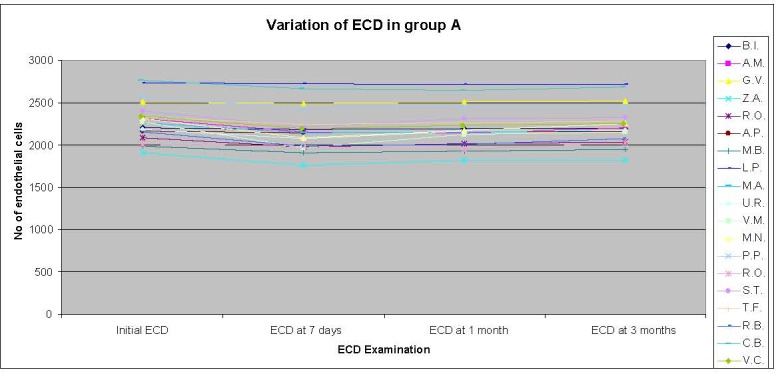
Variation of endothelial cells in group A

Preoperative ECD analysis revealed an average of 2290.21 cel/ mm2 +/ - 112.26 (95% confidence) (**Fig. 2**).

At 7 days, the ECD distribution was 2175.7895 ± 165.9038 (99% confidence) - a 6.99% loss of ECD, at 1 month it was 2207.7368 ± 155.4301 (99% confidence) and at 3 months it was 2285.9474 ± 179.8151.

We measured the mean pachymetry preoperatively at the level of 544.26 ± 23.15 microns. At 7 days it was 561 ± 36.62 microns, at 1 month it was 551 ± 28.16 microns and at 3 months it was 548 ± 26.75 microns.

The mean time of phacoemulsification was 3.4316 ± 0.7491 (99% confidence). For cataracts in the IV grade subgroup, the mean time was 5.5 ± 1.8392 (99% confidence). For those in the IIIrd subgroup, the mean time was 3.7556 ± 0.6431 (95% confidence), and for the subgroup II of hardness, the average time was 2.1286 ± 0.7593 (95% confidence). We weren’t able to find statistically significant differences between surgery performed by coaxial and bimanual techniques.

The results for lot B are detailed in **Table 2**.

**Table 2:** Patients in second group (B); Patients with * were operated by bimanual technique

Patient	Sex	Cataract grade	ECD initial	ECD at 7 days	ECD at 1 month	ECD at 3 months	EPT
AZ*	F	IV	2456	2216	2345	2311	5.6
VF	M	II	2784	2609	2657	2688	2.3
RG*	F	III	2136	1960	2024	2038	4.3
FJ*	M	III	2549	2387	2461	2472	3.9
TR	M	IV	2385	2199	2267	2272	3.9
SL*	F	II	2634	2588	2632	2629	1.8
PP	F	III	1875	1623	1650	1680	5.7
AP	M	IV	1937	1711	1756	1824	6.1
BM*	M	III	2372	2214	2218	2256	4.1
RT	F	II	2178	2094	2137	2139	1.3
SE*	M	II	2452	2333	2365	2342	2.6
UE	F	III	2528	2399	2413	2416	2.9
II*	M	III	2116	1967	2036	2057	3.8
IS*	M	IV	2368	1945	2056	2112	9.7
PR	F	IV	2356	2178	2224	2298	5.1
MG*	M	II	1845	1798	1870	1888	2.8
APC	F	III	2854	2569	2674	2733	3.8
VL*	M	III	2634	2527	2543	2531	2.9
IC	F	IV	2398	2215	2243	2248	4.6
NN*	M	II	2067	1984	1990	2032	1.8
CI*	M	IV	1953	1806	1840	1824	5.1
EG*	M	III	2356	2187	2197	2278	2.9
RR	M	II	2878	2812	2860	2853	1.5
TD	F	III	2639	2514	2528	2570	2.1
TN*	F	II	2379	2198	2386	2380	3.4
EB	M	IV	2428	2297	2314	2333	5.6
BM*	F	III	2534	2437	2475	2480	3.6
MS	M	II	1955	1894	1931	1933	2.2
ML	F	III	2614	2480	2497	2505	4.6
LS*	M	III	2333	2167	2272	2287	2.8
CC*	M	II	2167	2074	2098	2103	1.4
CF	F	III	2591	2386	2456	2481	3.6
PN*	F	IV	2871	2436	2610	2612	8.8
SM	M	III	2161	1986	2056	2098	4
RT*	F	II	2206	2145	2182	2195	2.6
TN	F	III	2284	2111	2125	2129	4.3
TL*	F	II	2378	2160	2204	2223	1.4
SP*	M	III	2446	2168	2241	2310	3.5
AI	F	III	2075	1889	1965	1991	3.4
IC	F	IV	2140	1816	1940	1952	5.2
EM	M	II	2056	2044	2038	2081	1.1
EN*	F	III	1945	1834	1870	1915	2.9
PD*	F	II	2916	2786	2821	2813	1.2
SP	F	III	2345	2187	2191	2222	3.3
PM*	M	III	2354	2176	2289	2310	3.4
MN	F	IV	2176	1945	1980	1974	5.6
NI	F	III	2482	2298	2331	2364	3.4
PP	M	II	2134	2097	2117	2131	1.3
ST*	F	II	2765	2658	2678	2681	1.9
PL*	F	III	2671	2531	2555	2551	3.7
MI	M	III	2137	1961	2072	2064	3.6

At 7 days, the ECD distribution was 2196 ± 104.3111 (99% confidence) - a 6.87% loss of ECD, at 1 month it was 2248.6275 ± 104.1418 (99% confidence) and at 3 months it was 2266.8431 ± 102.0706 (99%) – **Table 3**.

**Table 3:** ECD distribution at 7 days versus preoperative

Data Summary					
	A	B	Total		
n	51	51	102		
Σx	120293	111996	232289		
Σx2	287618165	249806276	537424441		
SS	3884716.980	3863060	8422680.990		
mean	2358.6863	2196	2277.3431		
Results					
Meana-Meanb	t	df	P	One-tailed	<.0001
162.6863	+13.66	50		Two-tailed	<.0001
	Observed	Confidence Intervals			
		0.95	0.99		
Meana	2358.6863	± 78.4523	± 104.6031		
Meanb	2196	± 78.2333	± 104.3111		
Meana-Meanb [Assuming equal sample variances]	162.6863	± 23.9363	± 31.9191		
Meana-Meanb [Assuming unequal sample variances	---	± ---	± ---		
Correlated Samples					

The mean pachymetry was preoperatively at 551.26 ± 28.75 microns. At 7 days it was 560.18 ± 34.26 microns, at 1 month it was 553.31 ± 29.38 microns and at 3 months it was 552.45 ± 28.89 microns.

The mean time of phacoemulsification was 3.5765 ± 0.6609 (99% confidence). For cataracts in the IVth subgroup, the mean time was 6.0556 ± 2.1625 (99% confidence). For those in the IIIrd subgroup, the mean time was 3.7556 ± 0.6431 (95% confidence), and for the subgroup of hardness II the average time was 2.1286 ± 0.7593 (95% confidence). We weren’t able to find a statistically significant difference between the technique of surgery with coaxial and bimanual.

The results that we obtain in **lot C** are here presented in **Table 4**.

**Table 4:** Patients in lot C. Patients with * were operated by bimanual technique

patient	sex	Cataract grade	ECD initial	ECD at 7 days	ECD at 1 month	ECD at 3 months	EPT
RG	F	III	2356	2116	2245	2311	5.6
PP*	M	II	2784	2711	2723	2756	1.3
RG*	F	III	2136	2086	2099	2118	3.3
LO	M	III	2349	2245	2276	2318	3.7
TR*	M	IV	2358	2199	2267	2298	4.9
LS*	F	II	2653	2588	2632	2629	1.8
PP	F	III	1975	1788	1845	1877	4.7
CA	M	II	1937	1888	1823	1903	2.1
MN*	M	III	2132	2014	2086	2145	4.1
TR	F	II	2095	2066	2088	2084	1.3
EU	M	II	2452	2333	2365	2342	2.6
BD*	F	III	2258	2119	2183	2193	2.9
MI*	M	III	2116	1967	2036	2057	3.8
GH	M	IV	2368	1945	2056	2112	7.7
CS*	F	II	2356	2178	2224	2298	5.1
CF*	M	II	1845	1798	1870	1888	2.8
MG	F	III	2854	2569	2674	2733	3.8
PI	M	III	2634	2527	2543	2531	2.9

At 7 days, the ECD distribution was 2174.2778 ± 188.3079 (99% confidence) - a 6.05% loss of EDC, at 1 month 2224.1667 ± 188.4944 (99% confidence) and at 3 months 2255.1667 ± 142.0706 (99%) as we presented it in **Table 5**.

**Table 5:** ECD distribution at 7 days versus preoperative

Data Summary					
	A	B	Total		
n	18	18	36		
Σx	41658	39137	80795		
Σx2	97801086	86384925	184186011		
SS	1390588	1290215.61	2857343.63		
mean	2314.3333	2174.2778	2244.3056		
Results					
Meana-Meanb	t	df	P	One-tailed	<.0001
140.0556	+6.01	17		Two-tailed	<.0001
	Observed	Confidence Intervals			
		0.95	0.99		
Meana	2314.3333	± 142.2398	± 195.4954		
Meanb	2174.2778	± 137.0102	± 188.3079		
Meana-Meanb [Assuming equal sample variances]	140.0556	± 49.1424	± 67.5416		
Meana-Meanb [Assuming unequal sample variances	---	± ---	± ---		
Correlated Samples					

The mean pachymetry was preoperatively at 551.26 ± 28.75 microns. At 7 days, it was 560.18 ± 34.26 microns, at 1 month it was 553.31 ± 29.38 microns and at 3 months it was 552.45 ± 28.89 microns.

The mean time of phacoemulsification was 3.5778 ± 1.113 (99% confidence). For cataracts in the IVth subgroup, the mean time was 6.3 ± 17.794 (99% confidence). For those in the subgroup III, the average time was 3.8667 ± 0.6657 (95% confidence), and for the subgroup II of hardness, the average time was 2.4286 ± 1.2164 (95% confidence). There were no statistically significant differences between patients operated by coaxial and bimanual techniques.

**Lot comparison**

By comparing lots A, B and C, the drop in EDC was relatively similar. We obtain a single statistical significance for a comparison regarding cataracts in the fourth lot of hardness, between lots A and C, at seven days after the presentation (p = 0.0472812).

## Conclusions

We observed a very big distributional variability when we speak about the structural and anatomical parameters of the eyeball itself and the level of cataract in the studied patients;

- cataract surgery performed by a phacoemulsification technique is a very safe and also an effective method, which is thought to be with good postoperative results;

- within each group, we observed a statistically significant decline in EDC when we speak about preoperative measurements, the one made at seventh day and the ones made at 1 month. This decrease was also statistically significant, and higher in patients with a grade IV cataract than in the ones with grade II or III cataract;

- this statistical significance was also observed in patients with grade IV and III, at the presentation of 3 months, while for grade II patients it wasn’t present;

- the mean time in regarding the phaco-emulsification in lot A was 3.4316 ± 0.7491 (99% confidence). For cataracts in the IV subgroup, the mean time was 5.5 ± 1.8392. For those in the subgroup III, the mean time was 3.7556 ± 0.6431 and for the subgroup II of hardness, the average time was 2.1286 ± 0.7593;

- the mean factor emulsification in lot B was 3.5765 ± 0.6609 (99% confidence). For cataracts in the IVth subgroup, the mean time was 6.0556 ± 2.1625. For those in the subgroup III, the mean time was 3.7556 ± 0.6431 and for the subgroup II of hardness, the average time was 2.1286 ± 0.7593;

- the mean factor emulsification in lot C was 3.5778 ± 1.113 (99% confidence). For cataracts in the IVth subgroup, the mean time was 6.3 ± 17.794. For the subgroup III hardness the average time was 3.8667 ± 0.6657 and for the subgroup II of hardness the average time was 2.4286 ± 1.2164. The high variability for the IVth group was in conjunction with the small size of the sublot - just 2 patients;

- we weren’t able to find a statistically significant difference when it comes with the actual time of mean phaco-emulsification between groups;

- also, we didn’t find a statistically significant difference when we speak about patients operated by coaxial and bimanual techniques;

- comparing lots A, B and C, the drop in EDC was relatively similar. The only comparison for which we obtained statistical significance was for cataracts in group IV of hardness, between lots A and C, at 7 days (p = 0.0472812);

- the conclusion of this study was that in cataract phacoemulsification surgery there was a statistical significance for the destruction of epithelial cells according to the ACD at cataracts in subgroup IV of hardness between patients with a small ACD and large ACD. For patients with severe cataracts and a small ACD, we need to pay more attention and to use viscoelastic adhesives in order to obtain an additional corneal protection;

- concerning the thing that in the rest of the analogies we didn’t find a statistical significance doesn’t mean that the difference does not exist. We accept the study’s limitations because we have to consider the relatively small size in terms of the lots and, of course, the unequal number of patients included the lots and, besides that, the fact that when we consider specular microscopy and the pachymetry – those were performed postoperatively at seven days after the surgery (when the patients are usually recovered);

- to obtain clearer results it should be necessary for us to study larger lots in order to carry out these measurements at 1st day post-operative and also at three days post-operative.

**Acknowledgements**

All authors had equal contribution.
